# Supplementary material for: The genome of Aechmea fasciata provides insights into the evolution of tank epiphytic habits and ethylene-induced flowering
Source: Commun Biol. 2022 Sep 7;5:920. doi: 10.1038/s42003-022-03918-4 (PMC9452560; doi:10.1038/s42003-022-03918-4)
Supplement: Supplementary file 2 — Supplementary Information [file 42003_2022_3918_MOESM2_ESM.pdf]

The genome of *Aechmea fasciata* provides insights into the evolution of tank epiphytic habits and ethylene-induced flowering

Zhiying Li[1,2,3,4,†], Jiabin Wang[1,2,3,4,†], Xuanbing Zhang[5], Guohong Zhu[5], Yunliu Fu[1,2,3,4], Yonglin Jing[1,2,3,4], Bilan huang[1,2,3,4], XiaoBing Wang[1,2,3,4], Chunyang Meng[1,2,3,4], Qingquan Yang[1,2,3,4] & Li Xu[1,2,3,4,*]

†These authors contributed equally to this work.
*Corresponding author
Email: xllzy@263.net

1 Institute of Tropical Crop Genetic Resources, Chinese Academy of Tropical Agricultural Sciences, Danzhou 571737, Hainan, China.

2 Ministry of Agriculture Key Laboratory of Crop Gene Resources and Germplasm Enhancement in Southern China, Danzhou, 571737, Hainan, China.

3 Hainan Province Key Laboratory of Tropical Crops Germplasm Resources Genetic Improvement and Innovation, Danzhou 571737, Hainan, China.

4 National Gene Bank of Tropical Crops, Danzhou, 571700, Hainan, China

5 College of Horticulture and Landscape Architecture, Hainan University, Haikou, 570228, China

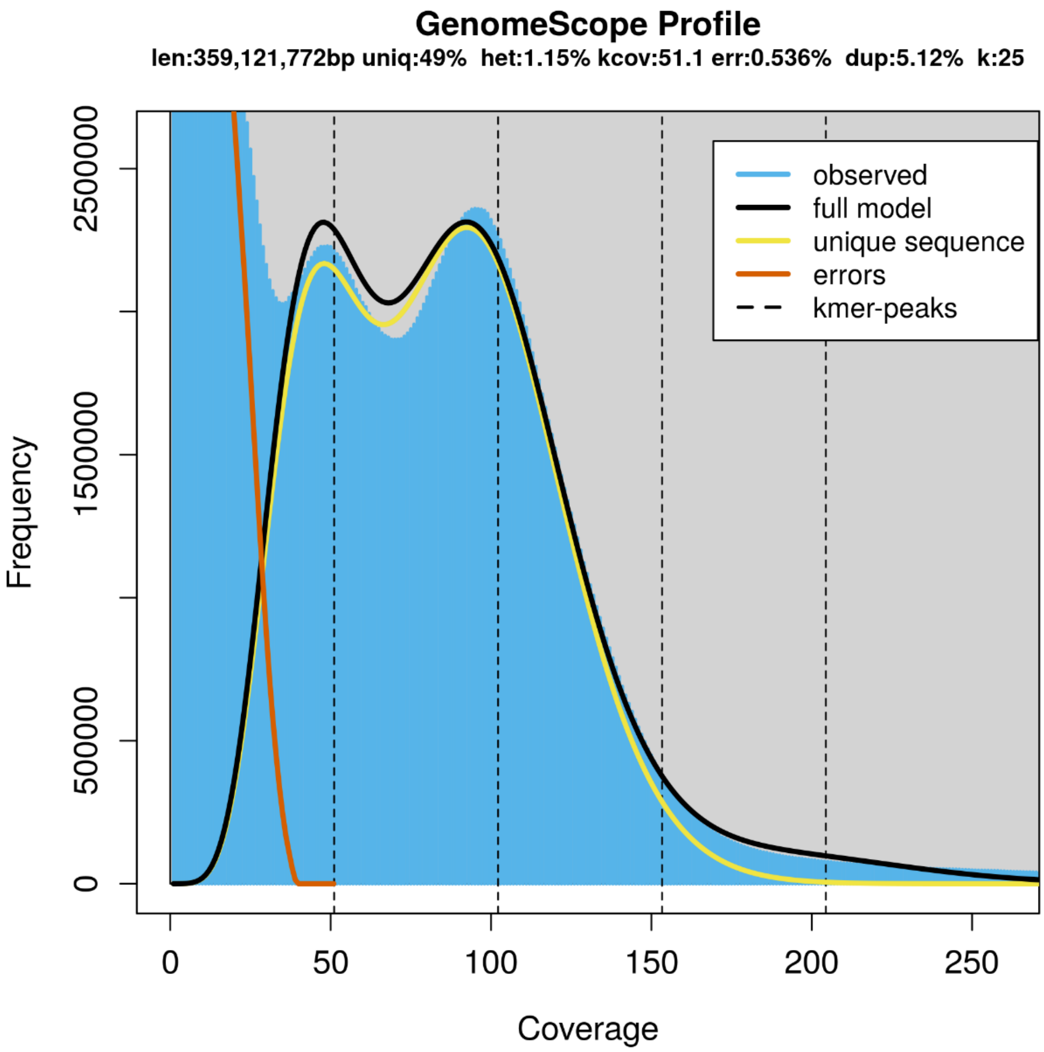

Supplementary Figure 1 Genome size estimation using GenomeScope.

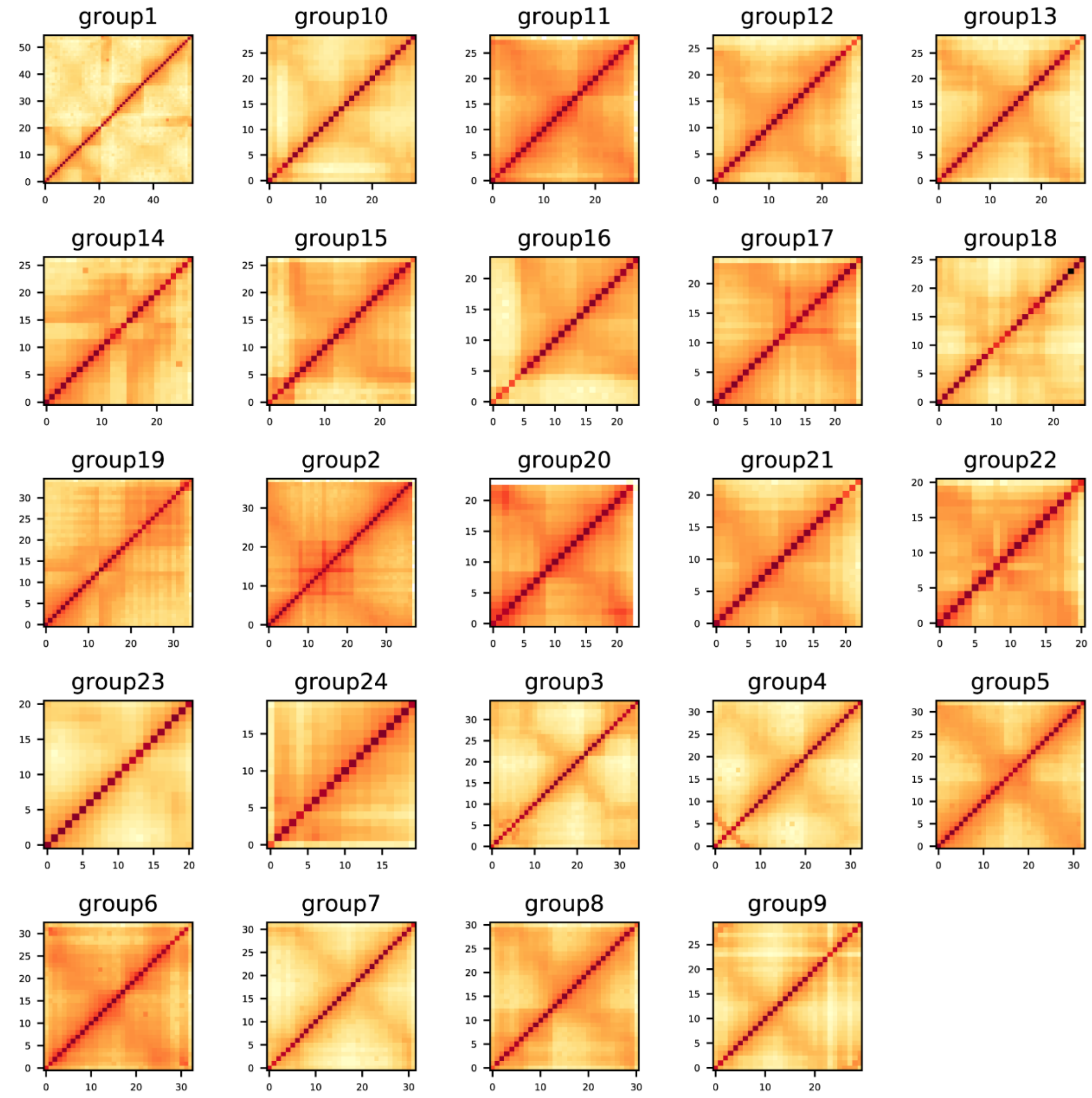

Supplementary Figure 2 Hi-C heatmap of each pseudo-chromosome.

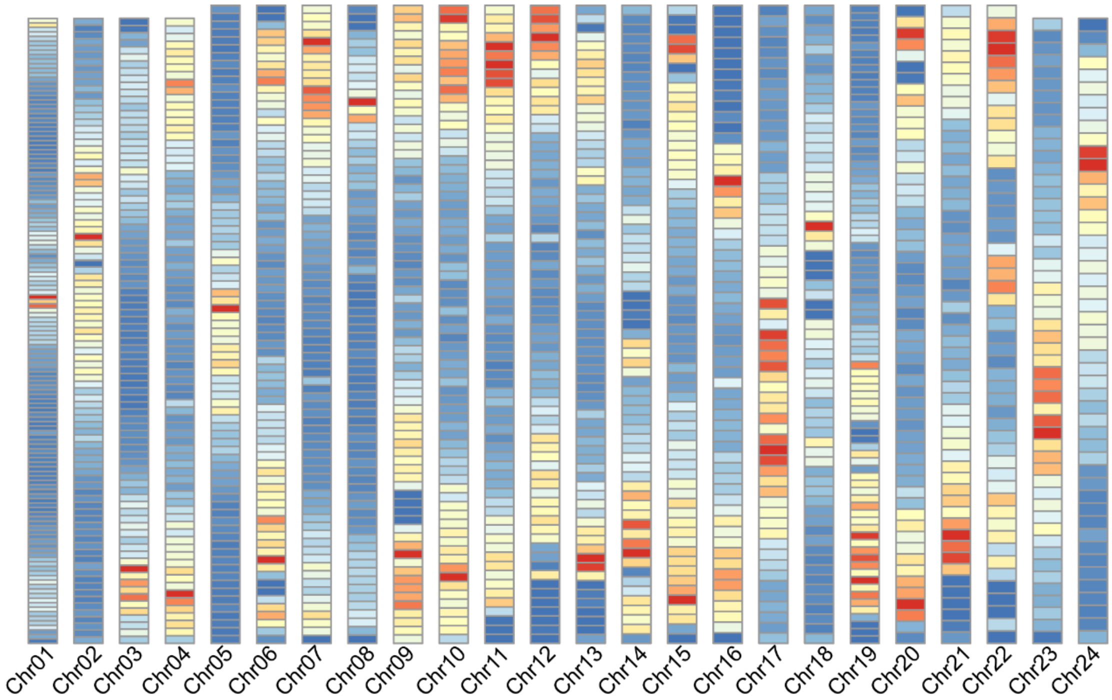

Supplementary Figure 3 Heat map of LTR distribution in each chromosome.

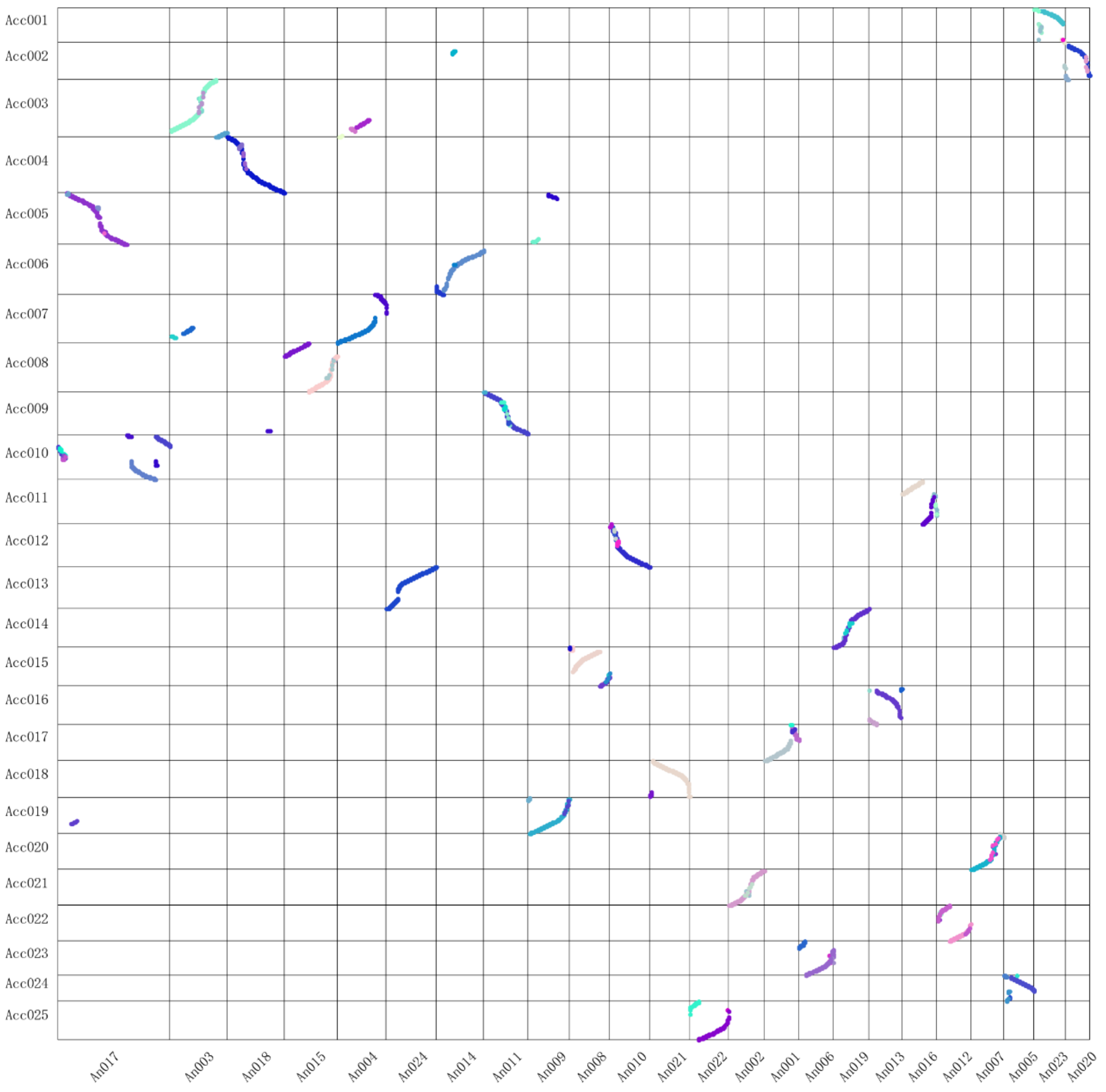

Supplementary Figure 4 Dot plot of synteny alignment between *A. comosus* and the ancestral genome.

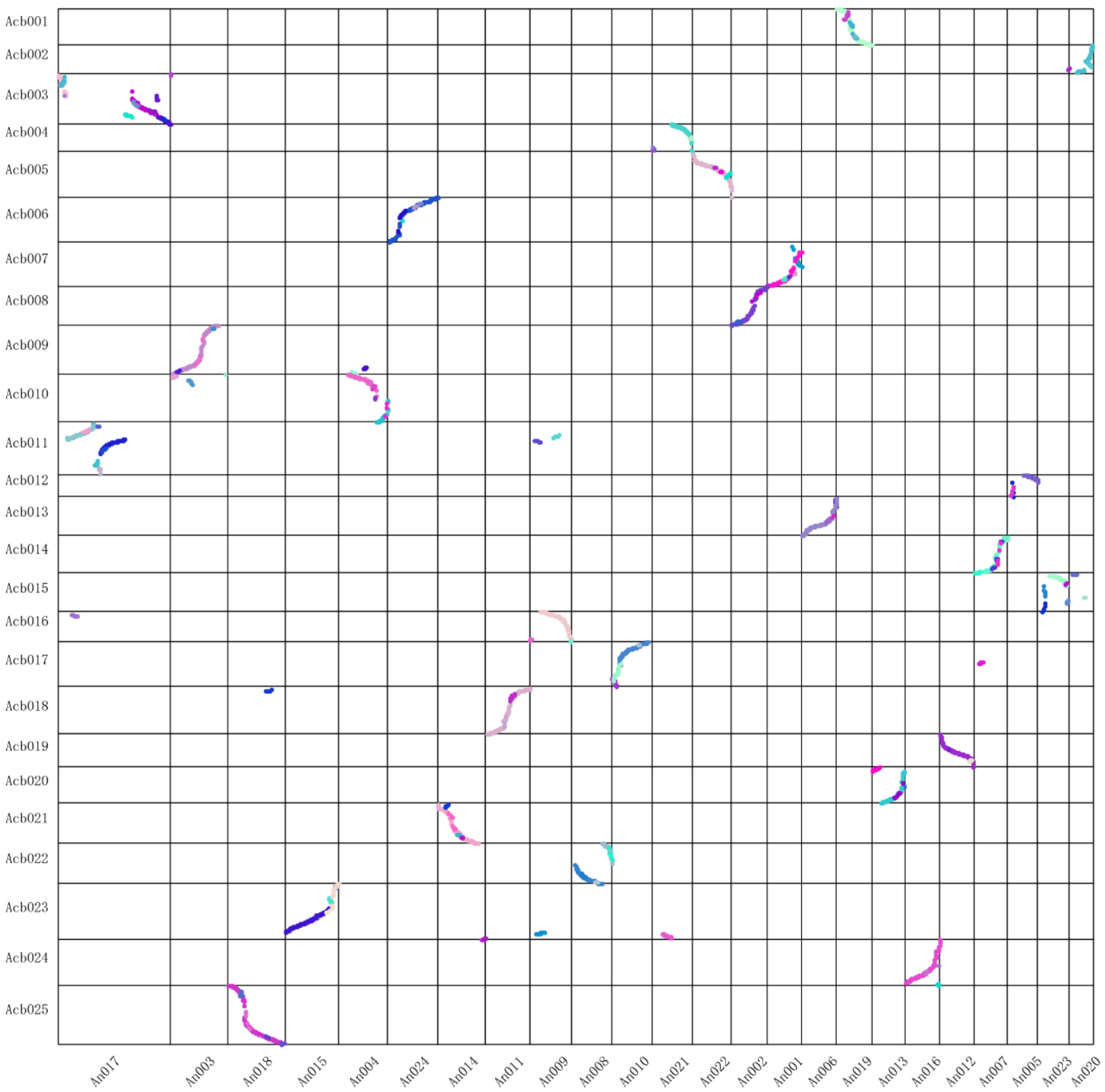

Supplementary Figure 5 Dot plot of synteny alignment between *A. bracteatus* and the ancestral genome.

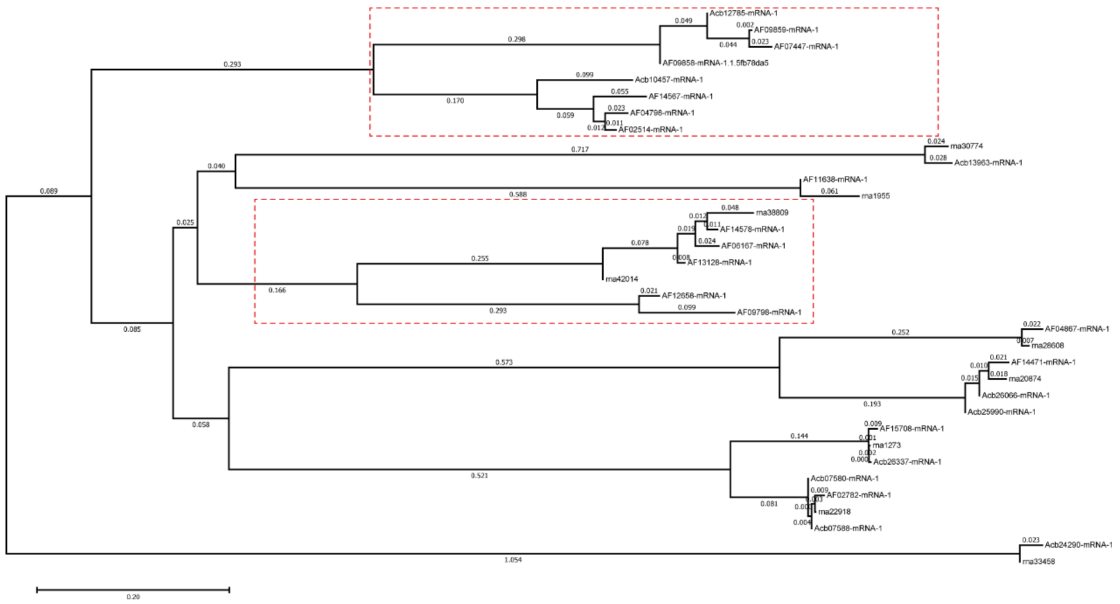

Supplementary Figure 6 Phylogenetic analysis of *Ricesleeper2* gene families.

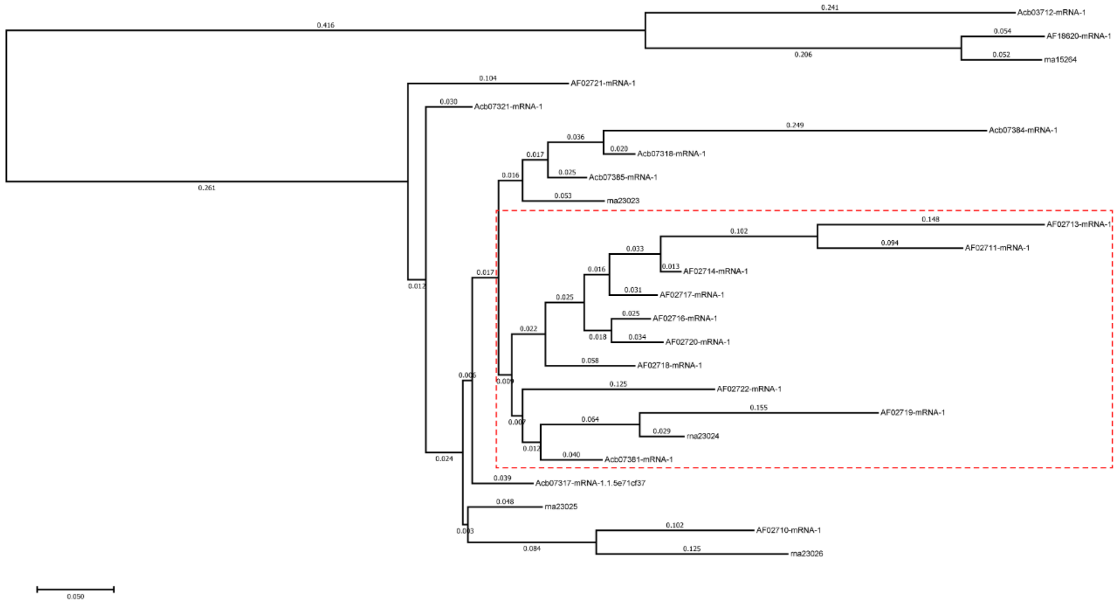

Supplementary Figure 7 Phylogenetic analysis of *TPS10* gene families.

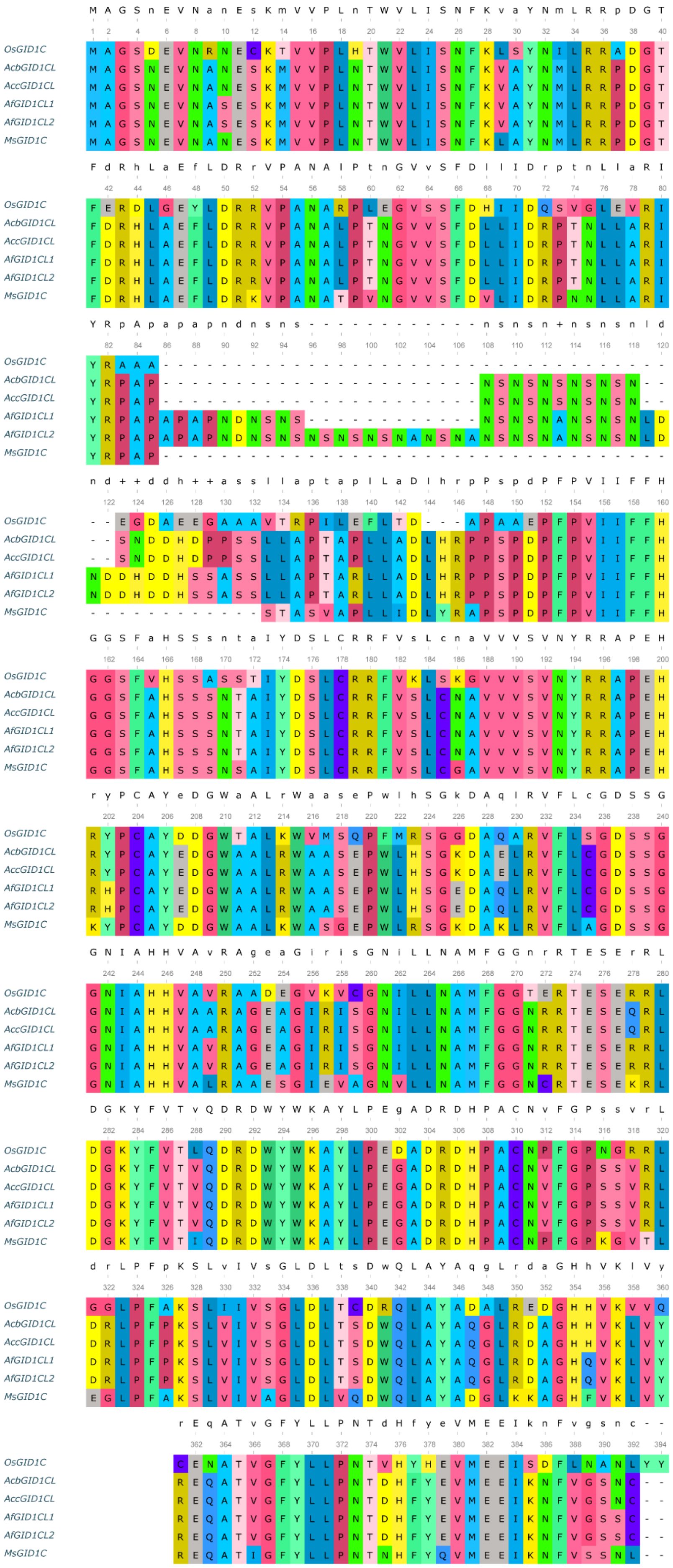
OsGID1C
AcbGID1CL
AccGID1CL
AfGID1CL1
AfGID1CL2
MsGID1C

Supplementary Figure 8 Multiple alignment of *GID1C* proteins. Os, *Oryza sativa subsp. Japonica*; Ms, *Musa acuminata subsp. malaccensis*; Acb, *A. bracteatus*; Acc, *A. comosus*; Af, *A. fasciata*.

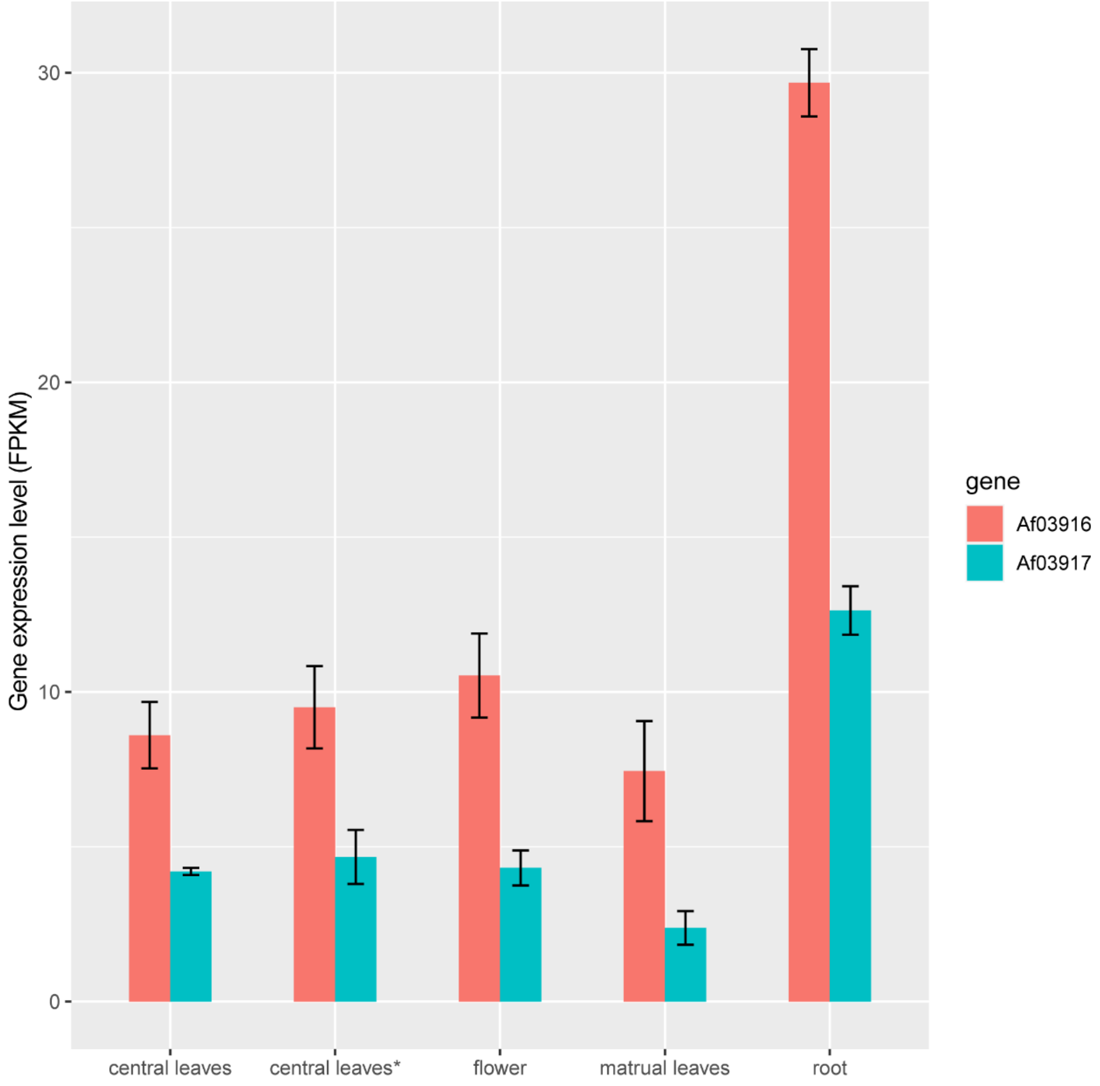

Supplementary Figure 9 Gene expression analysis of two *GID1C-like* genes in *A. fasciata*. Af03916, *AfGID1C-like1*; Af03917, *AfGDI1C-like2*.

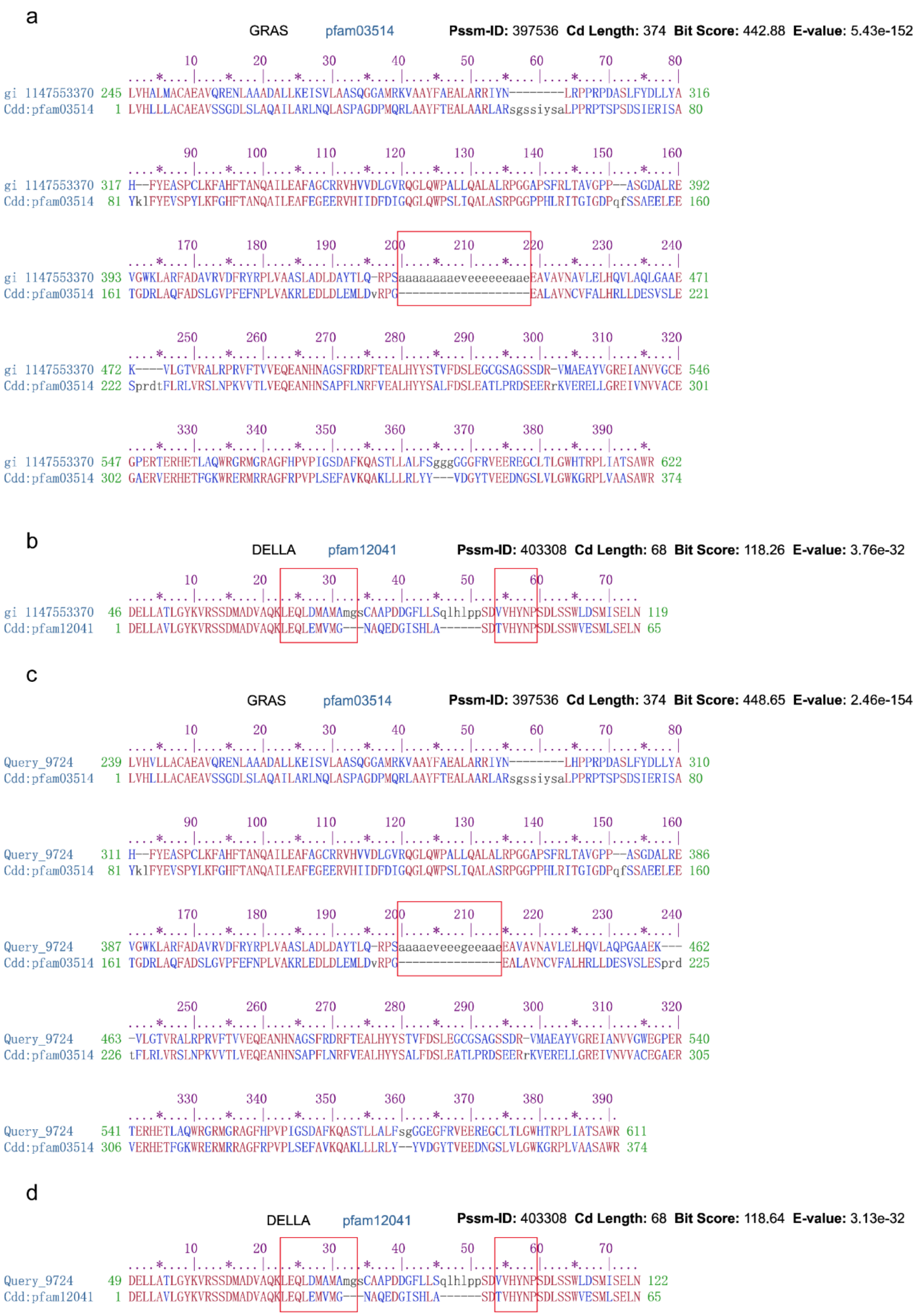

Supplementary Figure 10 Domain analysis of *AcSLR1-like* and *AfSLR1-like* based on searches of the NCBI Conserved Domain Database. a, alignment of *AcSLR1-like* at the GRAS domain; b, alignment of *AcSLR1-like* at the DELLA domain; d, alignment of *AfSLR1-like* at the GRAS domain; d, alignment

of *AfSLR1-like* at the DELLA domain.

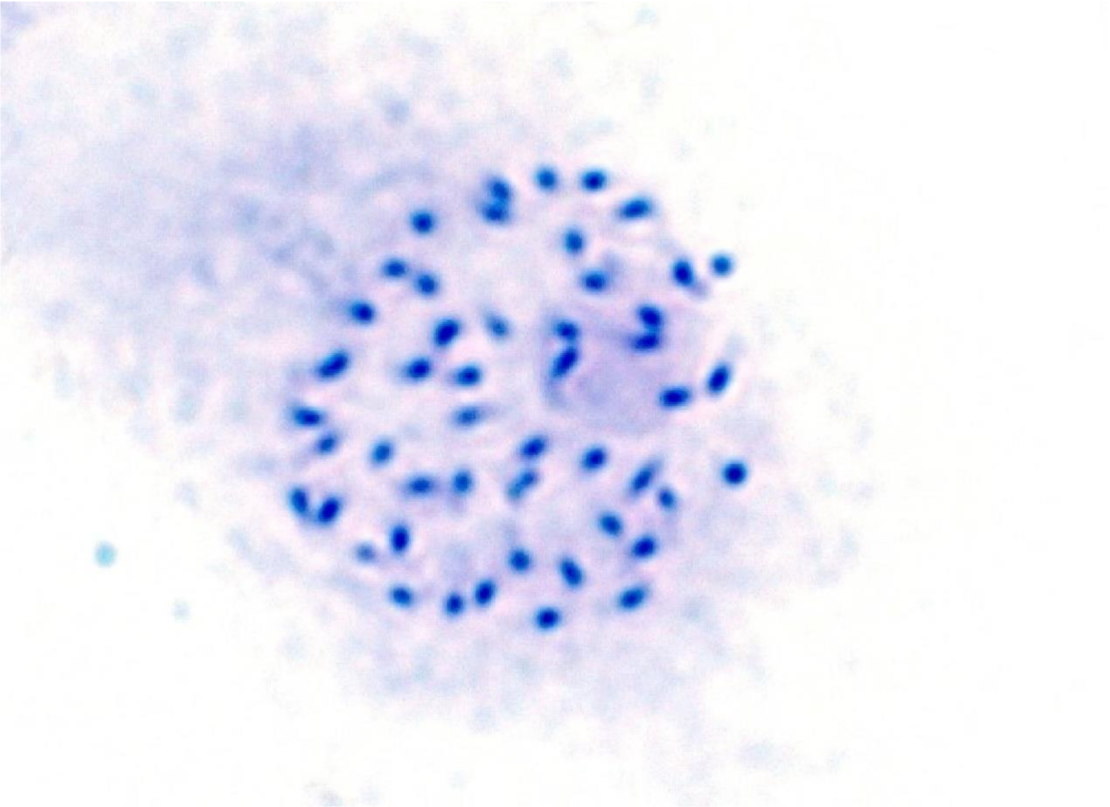

Supplementary Figure 11 Root tip chromosome karyotype analysis of *A. fasciata*.

Supplementary Table 1 Summary of genome assembly.

Type	Length (bp)	Count
N10	9050129	6
N20	7538727	12
N30	6962080	19
N40	5533891	28
N50	4689950	38
N60	2670604	53
N70	967756	86
N80	509414	166
N90	213451	329
Min.	45520	-
Max.	11307573	-
Ave.	792203	-
Total	529983447	669

Supplementary Table 2 Summary of protein-coding genes in *A. fasciata*.

Protein coding gene number	26,216
Total length of protein coding gene (bp)	108,991,579
Average length of protein coding gene (bp)	4157
Total exon length (bp)	28,099,890
Average length of exon (bp)	198
Total intron length (bp)	80891689
Average length of intron (bp)	712

Supplementary Table 3 Summary of BUSCO analysis of protein-coding genes from *A. fasciata* geome (93.4%).

397	Complete BUSCOs (C)
348	Complete and single-copy BUSCOs (S)
49	Complete and duplicated BUSCOs (D)
19	Fragmented BUSCOs (F)
9	Missing BUSCOs (M)
425	Total BUSCO groups searched

Supplementary Table 4 Summary of BUSCO analysis of *A. fasciata* geome (98.4%).

418	Complete BUSCOs (C)
410	Complete and single-copy BUSCOs (S)
8	Complete and duplicated BUSCOs (D)
5	Fragmented BUSCOs (F)
2	Missing BUSCOs (M)
425	Total BUSCO groups searched

Supplementary Table 5 Summary of repeat sequences in the *A. fasciata* genome.

		Number	Length (bp)	% of repeats	% of geneome
	**Repeats total**	499787	214719703	100	61.72471
LTR Retrotransposon	Gypsy	18790	83223889	38.75932	23.92408
	Copia	5445	13830252	6.441073	3.975734
	unknown	14837	18093919	8.426762	5.201395
Retrotransposon	LINE	5398	3349713	1.56004	0.9629301
	SINE	4	236	1.099107e-04	6.784208e-05
Unclassified	Unknown	143904	83787245	39.02168	24.08602

retroelement					
DNA transposon	Helitron	174	199246	0.09279353	0.05727654
	Academ-1	1	60	2.794341e-05	1.724799e-05
	MuLE-MuDR	921	1586369	0.7388092	0.4560279
	PIF-Harbinger'	123	100598	0.04685085	0.02891855
	hAT-Tip100	39	49081	0.02285817	0.01410914
	hAT-Tag1	114	249706	0.01162939	0.0717821
**TE total**		189750	204470314	95.22662	58.77836
	Simple_repeat	310037	10249389	4.773381	2.946356

Supplementary Table 6 ChIP-qPCR analysis of the binding of *AfEIL1-like* to *AfFTL2*.

Primer: 448-1: F, TAACGCACTCTCCCCTCAA; R, TCATTTATTCACGCCCTCTG.										
EXP		CT	AVE.	VOL(ul)	IDF	△CT	△CT（DNA normalized ）	%INPUT	ΔΔCt [ChIP/NIS]	Fold Enrichment
	IN 448-1	22.45	22.33	2	101	7.166666667	13.82487815	0.006891234	-2.05754719	4.16
		22.38								
		22.16								
	IP 448-1 EIN3	29.45	29.49666667	200						
		28.41								
		30.63								
	IgG 448-1		32.54	100	51	10.21	15.88242534	0.00165544		
		32.54								
	IN 448-1	22.45	22.33	2						
		22.38								
		22.16								

Primer: 448-2:F, TCAAACAAAGAAATACCCCG; R, CCAACGAATCTGACGGCAT.										
EXP		CT	AVE.	VOL(ul)	IDF	△CT	△CT（DNA normalized ）	%INPUT	ΔΔCt [ChIP/NIS]	Fold Enrichment
	IN 448-1	21.96	22.03666667	2	101	6.57	13.22821148	0.010421052	-2.07754719	4.22
		21.99								
		22.16								

	IP 448-1 EIN3	28.69	28.60666667	200						
		28.7								
		28.43								
	IgG 448-1	32.05	31.67	100	51	9.633333333	15.30575868	0.002468923		
		31.55								
		31.41								
	IN 448-1	21.96	22.03666667	2						
		21.99								
		22.16								
